# A role for hypoxia in craniofacial abnormalities

**Published:** 2013-07

Holoprosencephaly is a form of craniofacial abnormality that can result in miscarriage or stillbirth. Genetic and environmental factors are thought to play a role in the pathogenesis of holoprosencephaly and related birth defects, and recent studies have suggested that hypoxia (low oxygen conditions) might be associated with increased risk. In this study, Marcucio and colleagues explored the effects of a low-oxygen environment on craniofacial development in a chicken embryo model. Embryos exposed to hypoxia demonstrated a variety of craniofacial anomalies, including holoprosencephaly, and were less likely to survive. On a cellular level, hypoxia reduced cell proliferation and increased cell death (apoptosis). These data lend weight to the hypothesis that reduced oxygen conditions are detrimental during early development, and indicate that avoidance of factors that deplete oxygen levels during pregnancy, such as smoking, could reduce the occurrence of birth defects. **Page 915**

